# Geospatial Clustering of Mobile Phone Use and Tuberculosis Health Outcomes Among African Health Systems

**DOI:** 10.3389/fpubh.2021.653337

**Published:** 2022-02-18

**Authors:** Sunny Ibeneme, Nkiruka Ukor, Benson Droti, Humphrey Karamagi, Joseph Okeibunor, Felicitas Zawaira

**Affiliations:** ^1^World Health Organization, African Regional Office, Brazzaville, Republic of Congo; ^2^World Health Organization, Country Office, Abuja, Nigeria

**Keywords:** Africa, health systems, tuberculosis, mobile phone, differential local Moran's I

## Abstract

**Background:**

While multiple studies have documented the impacts of mobile phone use on TB health outcomes for varied settings, it is not immediately clear what the spatial patterns of TB treatment completion rates among African countries are. This paper used Exploratory Spatial Data Analysis (ESDA) techniques to explore the clustering spatial patterns of TB treatment completion rates in 53 African countries and also their relationships with mobile phone use. Using an ESDA approach to identify countries with low TB treatment completion rates and reduced mobile phone use is the first step toward addressing issues related to poor TB outcomes.

**Methods:**

TB notifications and treatment data from 2000 through 2015 that were obtained from the World Bank database were used to illustrate a descriptive epidemiology of TB treatment completion rates among African health systems. Spatial clustering patterns of TB treatment completion rates were assessed using differential local Moran's I techniques, and local spatial analytics was performed using local Moran's I tests. Relationships between TB treatment completion rates and mobile phone use were evaluated using ESDA approach.

**Result:**

Spatial autocorrelation patterns generated were consistent with Low-Low and High-Low cluster patterns, and they were significant at different *p*-values. Algeria and Senegal had significant clusters across the study periods, while Democratic Republic of Congo, Niger, South Africa, and Cameroon had significant clusters in at least two time-periods. ESDA identified statistically significant associations between TB treatment completion rates and mobile phone use. Countries with higher rates of mobile phone use showed higher TB treatment completion rates overall, indicating enhanced program uptake (p < 0.05).

**Conclusion:**

Study findings provide systematic evidence to inform policy regarding investments in the use of mHealth to optimize TB health outcomes. African governments should identify turnaround strategies to strengthen mHealth technologies and improve outcomes.

## Introduction

Mobile phones are becoming increasingly available and accessible globally. The global mobile phone subscription in 2009 was 68.0 per 100 inhabitants compared to 108 in 2019 corresponding to an overall 96% penetration rates^1^. In the African region, the estimated mobile phone penetration rate was 32.2% in 2008 compared to 85% in 2019, and is projected to rise to over 90% by 2025. The mobile broadband penetration rate also increased from 1.7% in 2008 to 30% in 2019 (1). Many people who could not access fixed telephones for health informatics now use the mobile phone technology to access health services (mHealth). Compared to the wired information technology, the wireless technology is less expensive, more convenient, and readily accessible for individuals in many developing countries, including African countries (2, 3). The mobile wireless technology has opportunities to facilitate communication among geographically isolated communities and could be harnessed to improve population health.

Mobile phones brought new opportunities for public health to Sub-Saharan Africa. With most of the African population in rural areas, the use of mobile phone has facilitated infectious disease management irrespective of the geographical barriers (4–6). Tuberculosis (TB) management is one area in which mobile phone use has shown great success because to effectively treat patients with TB, they must take four pills of anti-tuberculosis medications five times per week, for a period of 6 months (7). This may create a high level of non-compliance to prescribed medications. Such protracted adherence could be facilitated by removing barriers to access and utilization through mHealth technologies (8). In 2002, the South African government introduced the use of the mHealth technology and computer databases to optimize TB treatment adherence. The database repeatedly lists patients who are due for their medications and an automatic short-message-service (SMS) reminder is sent to their mobile phones. This model enhanced treatment adherence and completion rates among sampled patients (7). Thus, mobile phones provide platforms through which the SMS technology and other treatment-reminder protocols can be harnessed by patients with TB to improve efficiency and optimize outcomes (6, 8). Our study assumed that increased mobile phone use translates to broader access to TB services and represents the potential for impact on health with utilization of mobile phones to send/receive TB-related health information (9).

This study provides insight into the geospatial clustering of TB and mobile phone use among African health systems. Using an exploratory spatial data analytic (ESDA) approach, this study explored the spatial relationships between TB treatment completion rates and mobile phone use. Geospatial analytics of these concepts have opportunities to inform TB surveillance, intervention mapping and resource allocation. Moonan et al. (10) conducted a geospatial epidemiological TB surveillance among newly diagnosed patients with TB at the Tarrant County Health Department, Fort Worth, Dallas. Their model facilitated the identification of TB transmissions that were not identified during routine contact tracing, thereby enabling the identification of at-risk populations, with an intervention mapping recommended for screening, treatment, and rehabilitation (10).

Conceptually, mobile phones facilitate information exchange and transfer without spatial barriers at high efficiency and low cost (4, 5). Chadha et al. (11) evaluated the effectiveness of the ComCare mobile application in coordinating TB referrals among patients in the Khunti District of India. It was discovered that the mobile technology increased provider accountability and led to improved patient referral, retention, and treatment completion rates among network members (11). Other researchers showed similar successes demonstrating the use of geospatial analytics in TB control, prevention, and management. Mwila and Phiri (12) used geospatial analyses, cloud computing and web technologies to model TB prevention strategies among developing countries. They explored ways to optimize TB monitoring and tracking protocols using technologies that display geographic distribution of TB cases on an mHealth application, while providing policy reports to inform intervention mapping activities (12). While Yakam et al. (13) spatially identified smear-positive pulmonary TB clusters among poor neighborhoods in Douala, Cameroon using mHealth technologies, Herrero et al. (14) spatially explored cluster patterns of TB non-adherence and treatment dropouts in the metropolitan area of Buenos Aires, Argentina. Risk areas of non-adherence were characterized by poverty, ignorance, and reduced access to mHealth technologies (13, 14).

Multiple studies have documented the impact of mobile phone use on TB health outcomes for varied settings (11–14). However, the geospatial clustering patterns of TB treatment completion rates and mobile phone use among African countries is not immediately clear. Previous studies have focused on evaluating TB medication access using geospatial disaggregated datasets of population characteristics (13, 15). Hassarangsee et al. (16) investigated the spatial detection and management of TB using information systems in the Si Sa Ket Province, Thailand (16). However, the focus of this study is to evaluate the geospatial clustering patterns of TB treatment completion rates and mobile phone use among African health systems. It presents an exploratory spatial analysis on the relationships between TB treatment completion rates and mobile phone use for the countries in Africa. Using an ESDA approach to identify countries with low TB treatment completion rates and reduced mobile phone use could be the first step toward addressing issues related to poor TB outcomes. Thus, this presents an opportunity to identify African countries with limited resources and a high need for a wireless technology intervention.

## Materials and Methods

### Data Sources

Data^2^ for TB outcome and mobile phone use for African countries were obtained from the World Bank database for the periods from 2000 through 2015 (Supplementary Material A). Data collection is an annual event collected through an online-questionnaire sampling of government agencies. Non-responded questionnaires, including missing data are addressed by getting data from governments' websites and annual reports (17). This study excluded one country, South Sudan due to incomplete data. In total, 53 countries representative of the African continent were included in this study. Deidentified information was collated and aggregated per country and published at the end of each year by the World Bank, qualifying it as Institutional Review Board exempt (18).

### Comparative Statistical Analyses

*A*rcGIS (19) and GeoDa (20) statistical software were used in all geospatial analyses which were performed in three stages and completed in November 2020. Univariate local Moran's I and global Moran's I were run on TB treatment completion rates separately. This was followed by a differential local Moran's I analysis to ascertain differential cluster patterns for different time-periods. Finally, spatial relationships between TB treatment completion rates and mobile phone use for the year 2015 was evaluated using ESDA. To investigate treatment completion cluster patterns, spatial and tabular data were uploaded into ArcGIS 10.5.1. Geographically referenced data for TB treatment completion rates and mobile phone use for four time-periods (2000, 2005, 2010, and 2015) were extracted for each country. These were added and joined to the African map by country shapefile and analyzed using an ESDA approach to visualize patterns and trends among geographically referenced data.

The Local Indicator of Spatial Association (LISA) represents the localized equivalent of the global Moran's I (20, 21). For any location on the map, the LISA statistic measures and statistically tests the similarity of the geographically referenced data for that location (e.g., TB treatment completion rates at the source country) with the values of its corresponding local neighbors in space (surrounding countries). According to the standard practice for reporting geospatial analytics, positive spatial autocorrelation is placed into high-high and low-low clusters, and negative spatial autocorrelation is placed into high-low and low-high outliers. High-high clusters denote above-average values of core countries vs. surrounding countries. Low-low clusters represent below average values of core countries vs. surrounding countries. Low-High clusters mean small changes among core countries vs. high changes in the surrounding countries. Conversely, high-low clusters means high changes denotes core countries vs. small changes in the surrounding countries (22, 23). For this study, a randomization of 999 permutations was used prior to result interpretations, and this study only analyzed observations with neighbors (22, 23).

The local differential moran's I (LDMI) statistic measures if a variable change in space over time is related to its neighbors, and is calculated thus:


$$\begin{array}{l} I_{D,i}=v\left(y_{i,t}-y_{i,t-1}\right)\;\sum_{j}w_{ij}\left(y_{i,t}-y_{i,t-1}\right) \end{array}$$

Where y represents treatment completion rates for country *i* and neighbor *j*. The differential local Moran statistic *I*_*D*_ is generated based on change over time, and it is represented by the difference between *yt* and *yt-1*. The geospatial matrix (*W*_*ij*_) is a binary spatial weights matrix. Under the queen first order principle, contiguous geospatial neighbors with common borders and vertex weights equals one. Therefore, observations that share common borders are considered neighbors for the calculation, and all other locations are equal to zero (22, 23). LDMI determines spatial autocorrelation on change over time (yt - yt-1). For this study, differential cluster patterns were evaluated between base time 0 (year 2000) and time 1 (year 2005), time 2 (year 2010) and time 3 (year 2015), respectively. Year 2000 was chosen as the base time because of data availability which has been consistently captured for the 53 African countries included in this study (17). Also, notable access for free anti-tuberculosis medications commenced in 2000 among most African health systems (24).

## Results

**Figure 1:**
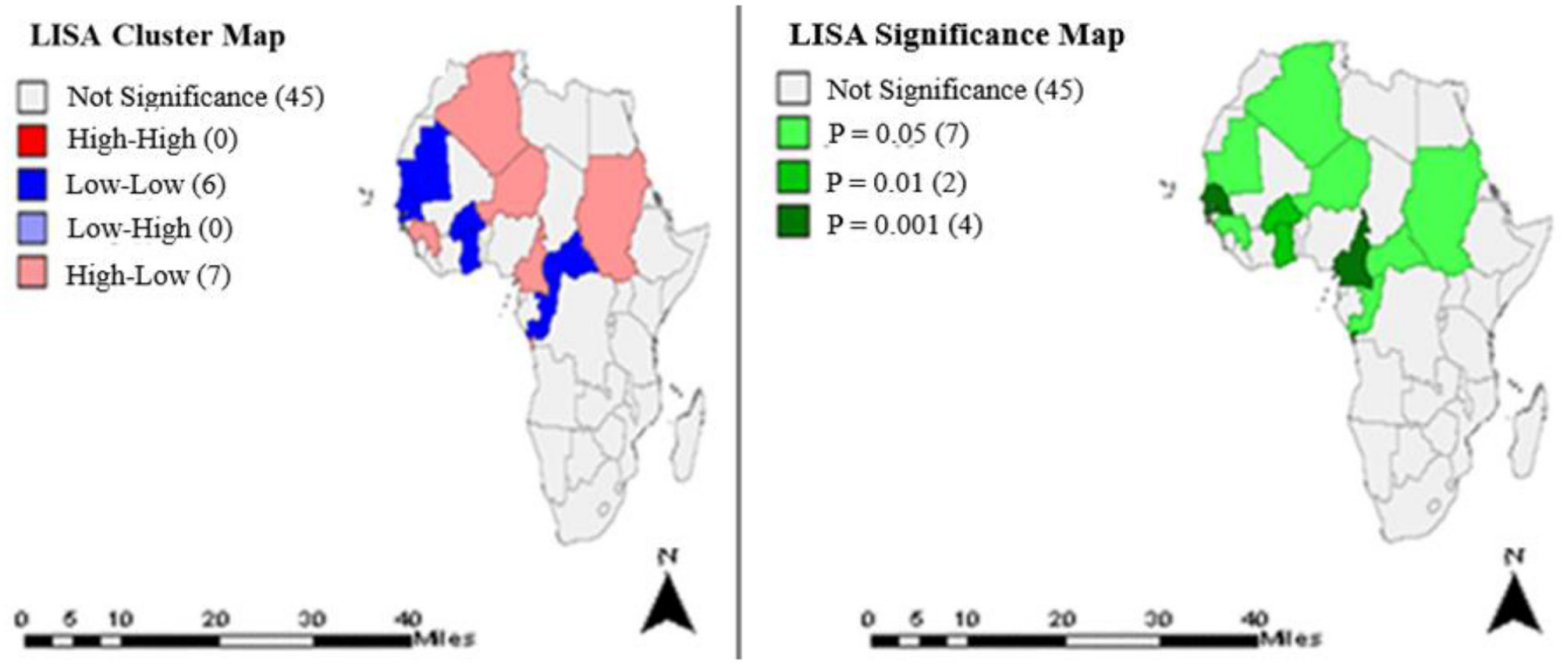
Clusters and significance levels of TB treatment completion rates in the year 2000.

**Figure 2:**
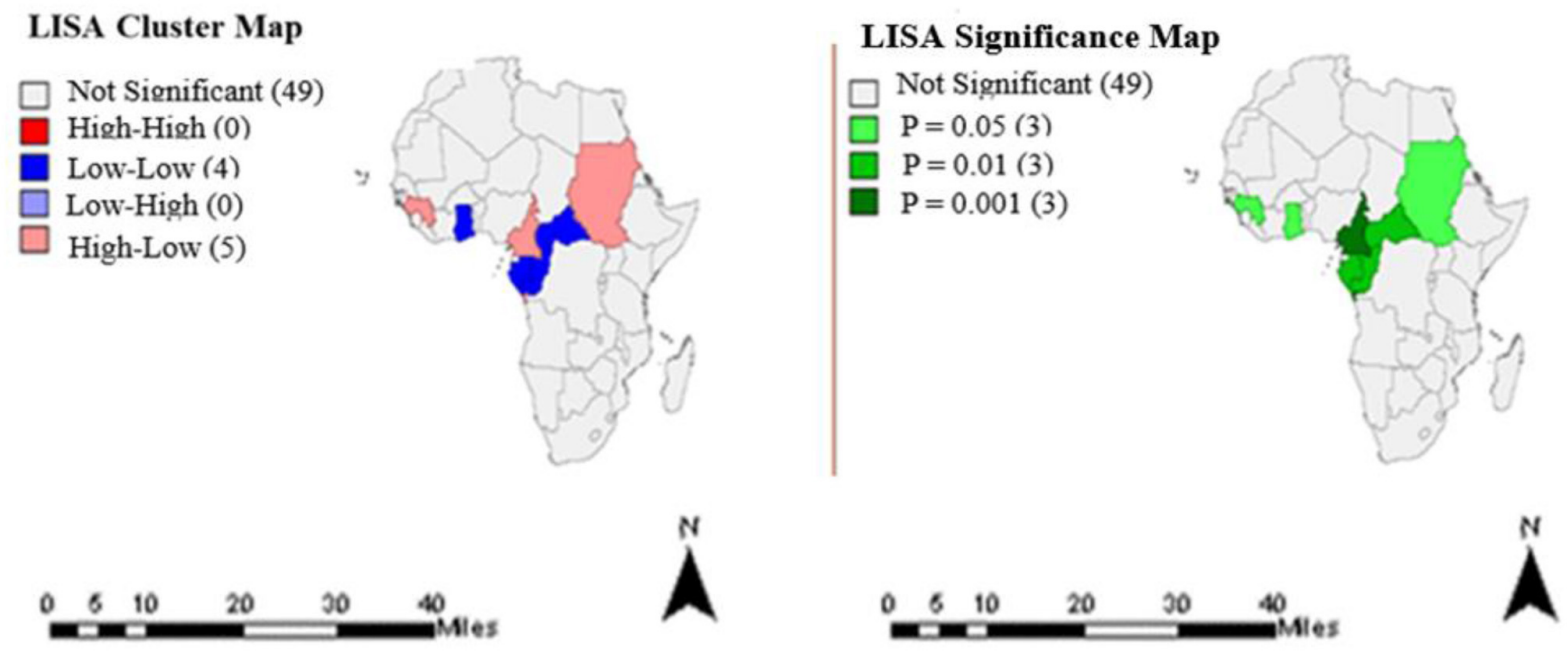
Clusters and significance levels of TB treatment completion rates in the year 2005.

**Figure 3:**
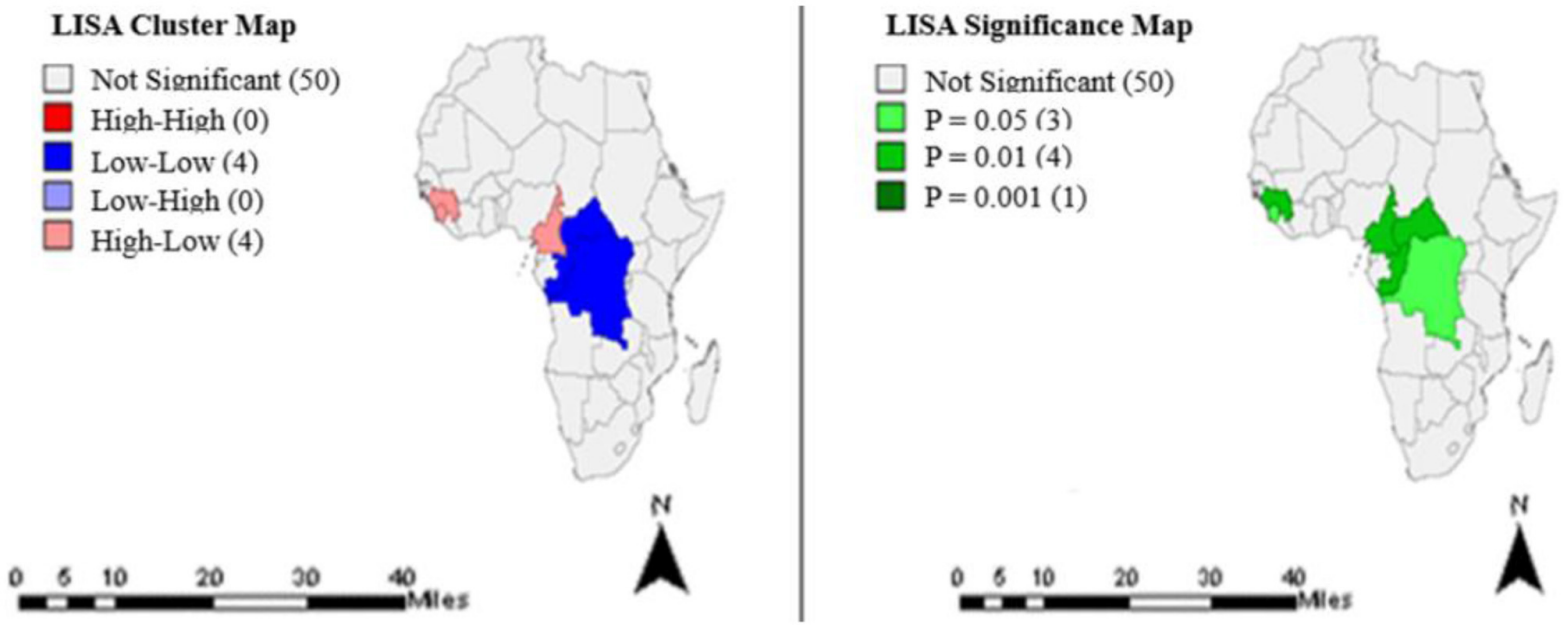
Clusters and significance levels of TB treatment completion rates in the year 2010.

**Figure 4:**
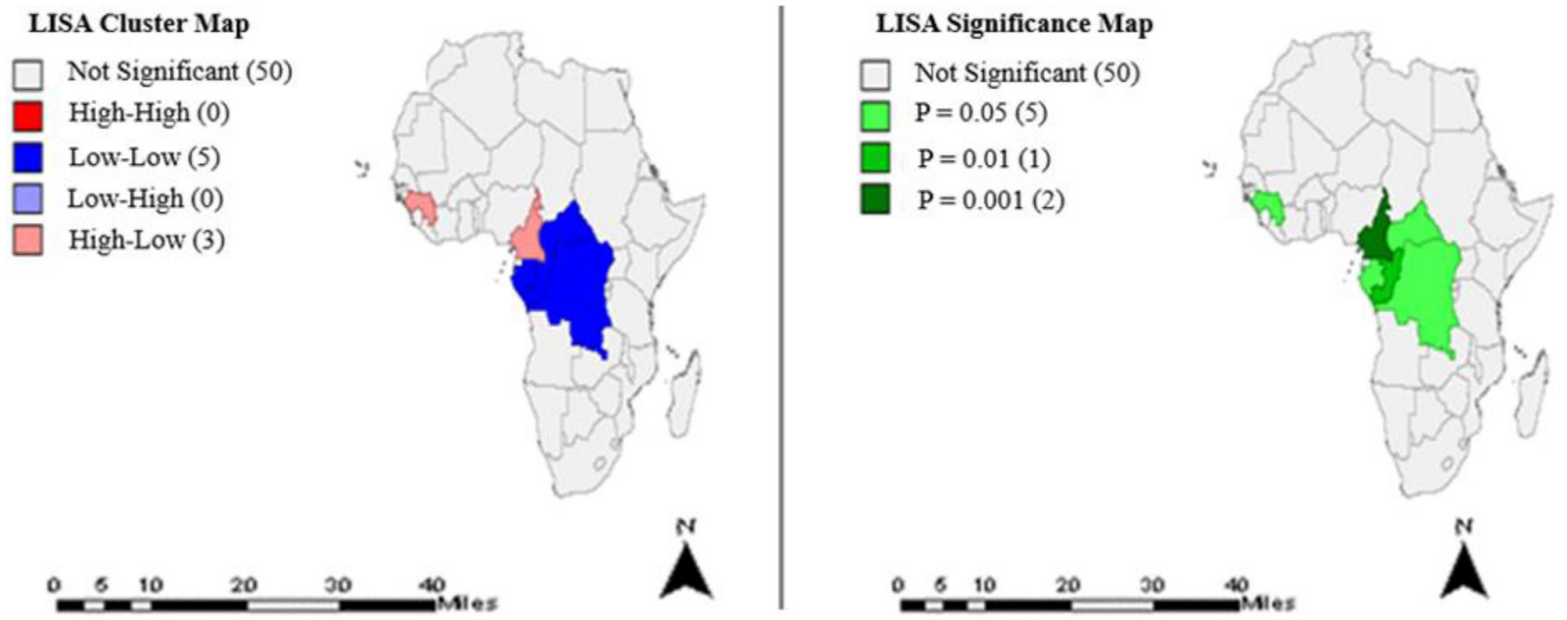
Clusters and significance levels of TB treatment completion rates in year 2015.

Using geospatial data for 53 African countries for the periods 2000–2015, univariate global Moran's I values and associated pseudo *p*-values were computed and documented (Supplementary Material B). In addition, LISA analytics identified different cluster pattern (low-low and high-low) that were significant at different *p*-values (Figures 1–**4**).

Figure 1 shows the clusters and significance levels of TB treatment completion rates in year 2000. Thirteen countries (Algeria, Mauritania, Niger, Burkina Faso, Togo, Ghana, Senegal, Gambia, Guinea, Sudan, Central Africa Republic, Cameroon, Congo) had significant clusters at different *p*-values.

Figure 2 represents the clusters and significance levels of TB treatment completion rates in year 2005. Nine countries had significant cluster patterns at different *p*-values.

The clusters and significance levels of TB treatment completion rates for year 2010 are shown in Figure 3. Eight countries had significant clusters at different *p*-values.

The cluster patterns and significance levels of TB treatment completion rates for year 2015 are shown in Figure 4. Eight countries had significant clusters at different *p*-values.

### LDMI Analyses

The local differential Moran's I analytics identified different cluster patterns for the different time periods evaluated (Tables 1–**3**).

**Table 1:** Differential Local Moran's I estimations of TB treatment completion rates between time 0 (2000) and time 1 (2005).

**Variable: TB_2000 and 2005**	**Countries**	**Cluster type**
	Algeria	High-Low^*^
	Burkina Faso	Low-Low^*^
	Senegal	Low-Low^**^

*Significance level*:

* *p < 0.05*;

** *p < 0.01*.

Table 1 represents LDMI results between time 0 (2000) and time 1 (2005). Algeria, Burkina Faso, and Senegal had significant clusters at different *p*-values (Supplementary Material C).

Table 2 shows the result between time 0 (2000) and time 2 (2010). Niger, Senegal, Gambia, Namibia, Lesotho, Djibouti, Algeria, Cameroon, South Africa, Democratic Republic of Congo (DRC), Kenya, and Sierra Leone had significant clusters at different *p*-values (Supplementary Material C).

**Table 2:** Differential Local Moran's I estimations of TB treatment completion rates between time 0 (2000) and time 2 (2010).

**Variable: TB_2000 and 2010**	**Countries**	**Cluster type**
	Niger	Low-Low^*^
	Senegal	Low-Low^**^
	Gambia	Low-Low^**^
	Namibia	Low-High^*^
	Lesotho	Low-High^*^
	Djibouti	Low-High^*^
	Algeria	High-Low^*^
	Cameroon	High-Low^*^
	South Africa	High-Low^*^
	Dem Rep Congo	High-High^*^
	Kenya	High-High^*^
	Sierra Leone	High-High^*^

*Significance level*:

* *p < 0.05*;

** *p < 0.01*.

Table 3 represents estimation results between time 0 (2000) and time 3 (2015). Niger, Burkina Faso, Senegal, South Africa, Algeria, Cameroon, and DRC had significant clusters at different *p*-values (Supplementary Material C).

**Table 3:** Differential Local Moran's I estimations of TB treatment completion rates between time 0 (2000) and time 3 (2015).

**Variable: TB_2000 and 2015**	**Countries**	**Cluster type**
	Niger	Low-Low^*^
	Burkina Faso	Low-Low^*^
	Senegal	Low-Low^***^
	South Africa	Low-Low^**^
	Algeria	High-Low^**^
	Cameroon	High-Low^**^
	Dem Rep Congo	High-High^**^

*Significance level*:

* *p < 0.1*;

** *p < 0.05*;

*** *p < 0.01*.

### Spatial Correlation Analyses

**Figure 5:**
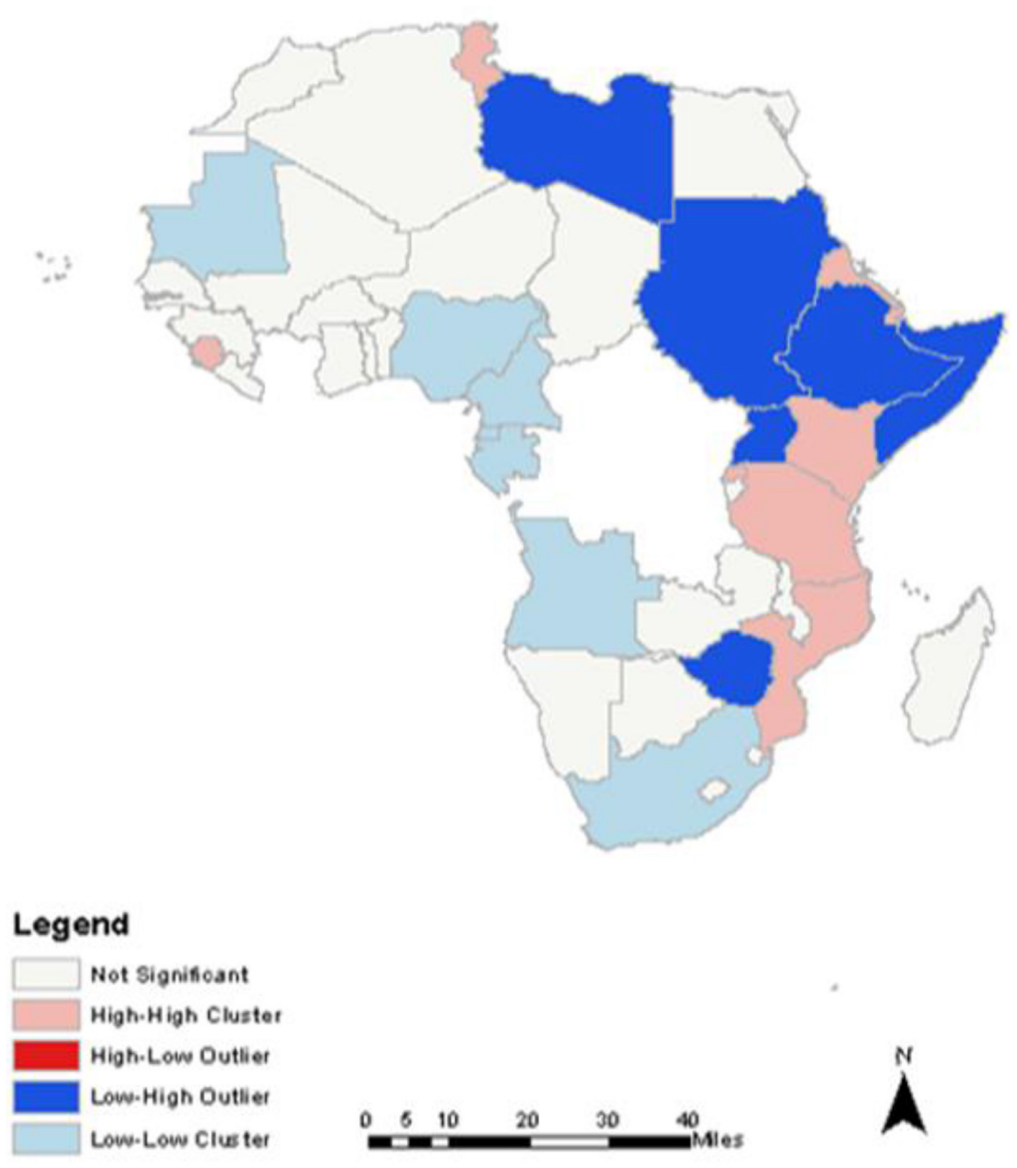
Spatial Correlation result between TB treatment Completion Rates and Mobile phone use.

Spatial relationship between TB treatment completion rates and mobile phone use identified high-high clusters, low-low clusters, and low-high outliers (Figure 5).

## Discussions and Policy Implications

Exploratory spatial data analysis identified statistically significant clusters in TB treatment completion rates among some countries of Africa. Most African countries except those in the Southern region had significant clusters (Figures 1–4), predominantly low-low clusters (14 countries) and high-low clusters (10 countries). Low-Low clusters suggest that TB treatment completion rates between 2000 and 2015 were low among identified countries and were surrounded by countries with similar low changes. High-low clusters suggest high changes in the core countries vs. low changes in their surrounding countries (19, 20). In the year 2000, Algeria had a “High-low” cluster tract (Figure 1). This suggests that the TB treatment completion rates for Algeria in 2000 was high, surrounded by countries with low rates. DRC had a “low-low” cluster tract in year 2010, suggesting that TB treatment completion rates were low for DRC, surrounded by countries with similar low changes (Figure 3). These findings could inform the formation of health policies for TB management strategies including resource allocation frameworks in countries. Study results also indicated that only a few countries had treatment completion rates for TB within the period of this study analyses (Figures 1–4): with all clusters identified either as outliers or cold spots, and 13 countries out of 53 had treatment completion rates in the year 2000 (Figure 1). However, treatment completion rates in 2005, 2010, and 2015 were nine countries (Figure 2), eight countries (Figure 3) and eight countries (Figure 4), respectively. These suggest that poor compliance and non-adherence could impact treatment completion rates for which the mHealth technology could be harnessed to address issues related to access, utilization, and attrition (8, 14).

Differential Moran's I cluster maps identified hot spots, cold spots, and outliers among African countries. The base-case analysis identified three countries with significant clusters including Algeria, Burkina Faso, and Senegal. Cluster patterns for Burkina Faso and Senegal were low-low cluster tracts, which suggest low TB treatment completion rates surrounded by countries with similar low changes. Conversely, Algeria had high-low clusters suggesting high changes in TB treatment completion rates vs. low changes in the surrounding countries (Supplementary Material C, Supplementary Figure 1). These findings could inform planning and suggest that the mHealth technology have opportunities to improve outcomes by facilitating fast, reliable, and updated health information.

While the time-period 2 analysis identified 12 countries with High-High clusters (Supplementary Material C, Supplementary Figure 2), the time-period 3 analysis identified eight countries with significant clusters (Supplementary Material C, Supplementary Figure 3). Altogether, two countries including Algeria and Senegal had significant clusters across the three time-periods of study evaluation (Supplementary Material C). Nonetheless, of note is the fact that only three countries had significant clusters from LDMI analytics in the year 2005 (Supplementary Material C, Supplementary Figure 1), compared to 2010 and 2015, where 12 countries (Supplementary Material C, Supplementary Figure 2) and seven countries (Supplementary Material C, Supplementary Figure 3) had significant clusters, respectively. A possible explanation could be that the 5-year period may not be enough time to appreciate significant changes in TB treatment rates (24). Conversely, the 15-year period may be too long to observe this trend. Thus, it appears the ideal time for this evaluation should be around 10-year time-periods. This gives credence to treatment guideline policy change introduced in 2009 by WHO that advocates following patients post treatment for longer periods irrespective of the form of TB (24). In view of this, future studies should aim to do a sensitivity analysis to determine the precise time to explore TB treatment completion rates among African health systems.

It must be noted that identifying significant clusters in TB treatment completion rates in any country does not translate to TB-free nations. South Africa is one of the nations with good TB programs in Africa (25), corroborating this study findings (Tables 1–3). South Africa had significant clusters for TB treatment completion rates (Supplementary Material C), which could possibly be attributed to the coordinated TB control measures introduced by the South African government (7). However, despite government efforts to curb the incidence of TB in South Africa, a recent study by the WHO identified South Africa as one of the seven countries that accounted for 64% of global new TB cases (25). Thus, notwithstanding significant TB treatment completion clusters identified by this study, the burden of TB in South Africa remains high.

Exploratory spatial data analyses identified significant association between TB treatment completion rates and mobile phone use rates. Countries with higher rates of mobile phone use showed higher TB treatment completion, rates suggesting enhanced program uptake. Dissecting this association with local level geographical data revealed differing cluster patterns, suggesting that the diffusion was not consistent across the region.

High-high clusters indicate countries with high TB treatment completion rates surrounded by countries with similar high mobile phone use rates. Tunisia, Sierra Leone, Eritrea, Kenya, Rwanda, Tanzania, and Mozambique had high-high clustering of these two attributes (Figure 5). This suggests that the use of mobile phones may be facilitating TB treatment completion rates; and gives credence to findings by Chadha et al. (11) who demonstrated how the mobile phone technology strengthened and optimized TB health outcomes (11).

Low-low clusters indicate countries with low TB completion rates surrounded by countries with low mobile phone use rates. Mauritania, Nigeria, Cameroon, Equatorial Guinea, Gabon, Angola, and South Africa had significant low-low clustering of these two attributes (Figure 5). Such low uptakes may contribute to the high burden of TB in Nigeria and South Africa and lends credence to reports by WHO that listed them among the six countries that contributed to the high burden of global new cases of TB in 2017 (26).

Spatial outliers indicate countries with either high or low TB completion rates surrounded by countries with either low or high mobile phone use rates. Identified low-high outliers include Zimbabwe, Somalia, Ethiopia, Libya, Sudan, and Uganda (Figure 5). These are potential moderators and mediators for this study, and could possibly be related to factors that impede the use of mobile phones for health informatics including poverty, ignorance, and poor access to mHealth technologies (14).

This study had some limitations. It did not control for the presence or absence of factors that could influence access and utilization of services, which could impact the robustness of the study findings. In addition, there were some limitations in the datasets used for this study. Geographical data for TB cases from the World Bank were at the country level only, and granular spatial relationships could have been used and would have revealed cases at a finer resolution. More so, this study is exploratory in nature. It assesses correlation and not causation and becomes the first step in assessing the relationship between TB health outcomes and potential impacts of ICT tools such as mobile phone use on TB programs among African health systems.

## Conclusion

Exploratory spatial data analyses identified positive spatial autocorrelation for the periods evaluated, as well as varying cluster patterns of TB treatment completion rates across the periods of study evaluation. There was a direct spatial relationship between TB treatment completion rates and mobile phone use among related African countries. Spatial autocorrelation patterns generated were consistent with low-low and high-low cluster patterns and were significant at different *p*-values. Algeria and Senegal had significant clusters across the study periods, while DRC, Niger, South Africa, and Cameroon had significant clusters in at least two time-periods. ESDA identified statistically significant associations between TB treatment completion rates and mobile phone use. Countries with higher rates of mobile phone use showed higher TB treatment completion rates overall, indicating enhanced program uptake. Thus, there is a need to strengthen national policies that promote TB medication adherence and completion using mHealth strategies among African health systems. African government should identify turnaround strategies to strengthen mHealth technologies and improve health outcomes.

## Data Availability Statement

The original contributions presented in the study are included in the article/Supplementary Material, further inquiries can be directed to the corresponding author/s.

## Author Contributions

SI, NU, and BD conceived, coordinated, wrote the first draft of the manuscript, and did the final review and edit of the draft manuscript. HK, JO, and FZ participated in the study conception and overall study coordination and contributed to writing the subsequent drafts of the manuscript. All authors have read and approved the final draft manuscript before publication.

## Conflict of Interest

The authors declare that the research was conducted in the absence of any commercial or financial relationships that could be construed as a potential conflict of interest.

## Publisher's Note

All claims expressed in this article are solely those of the authors and do not necessarily represent those of their affiliated organizations, or those of the publisher, the editors and the reviewers. Any product that may be evaluated in this article, or claim that may be made by its manufacturer, is not guaranteed or endorsed by the publisher.

## Footnotes

^1^Mobile phone penetration rate is the degree of diffusion of mobile phone use.

^2^Tuberculosis treatment completed rate (% of new cases) and Mobile-cellular telephone subscriptions per 100 inhabitants.

## Abbreviations

SMS: Short Message Services
TB: Tuberculosis
WHO: World Health Organization
mHealth: Mobile Health
ICT: Information and Communication Technology
ESDA: Exploratory Spatial Data Analytic
LISA: Local Indicator of Spatial Association
LDMI: Local Differential Moran's I.
