# Supplementary material for: Geospatial Clustering of Mobile Phone Use and Tuberculosis Health Outcomes Among African Health Systems
Source: Front Public Health. 2022 Feb 18;9:653337. doi: 10.3389/fpubh.2021.653337 (PMC8895232; doi:10.3389/fpubh.2021.653337)
Supplement: Supplementary file 1 [file Data_Sheet_1.docx]

**Supplementary Material – A**

African countries used in this analysis as listed by the World Health Organization. Available at:<http://www.who.int/countries/en/> accessed April 2, 2020.

Algeria, Burundi, Burkina Faso, Benin, Angola, Cameroon, Botswana, Cape Verde, Central African Republic, Comoros, Chad, Congo, Democratic Republic of Congo, Côte d'Ivoire, Djibouti, Equatorial Guinea, Egypt, Eritrea, Ethiopia, Gabon, Ghana, Guinea-Bissau, Lesotho, Gambia, Guinea, Kenya, Liberia, Libya, Malawi, Mauritania, Mali, Madagascar, Mauritius, Mozambique, Morocco, Nigeria, Namibia, Niger, Sao Tome and Principe, Seychelles, Rwanda, Senegal, Sierra Leone, Somalia, South Africa, South Sudan, Sudan, Swaziland, Togo, Uganda, Tunisia, Tanzania, Zambia, Zimbabwe.

**Supplementary Material – B**

**Univariate global Moran’s I result for the years 2000 – 2015.**

|  | **Variables** | **Moran’s I value** | **Pseudo p-value** |
| --- | --- | --- | --- |
|  | **TB_TreatmentCompletionRate_Yr2000** | **0.0190** | **0.021** |
| **Univariate Global Moran’s I** | **TB_TreatmentCompletionRate_Yr2005** | **0.0177** | **0.032** |
|  | **TB_TreatmentCompletionRate_Yr2010** | **0.0196** | **0.025** |
|  | **TB_TreatmentCompletionRate_Yr2015** | **0.0179** | **0.031** |

**Supplementary Material – C**

| **Figure 1: Differential Local Moran’s I estimations of TB treatment completion rates between time 0 (2000) and time 1 (2005)**  **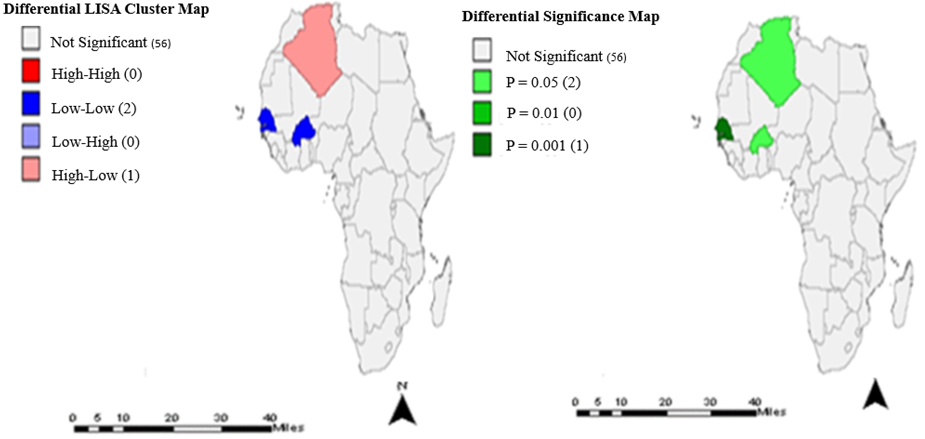** |
| --- |
| **Figure 2: Differential Local Moran’s I estimations of TB treatment completion rates between time 0 (2000) and time 2 (2010)**  **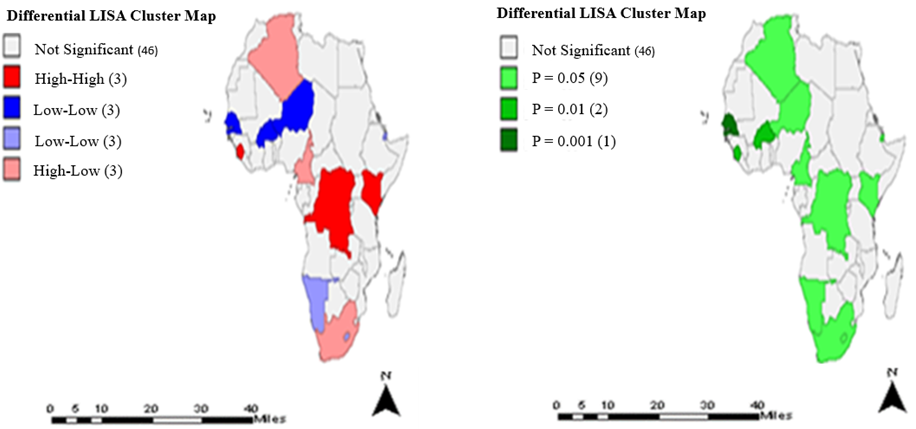** |
| **Figure 3: Differential Local Moran’s I estimations of TB treatment completion rates between time 0 (2000) and time 3 (2015)**  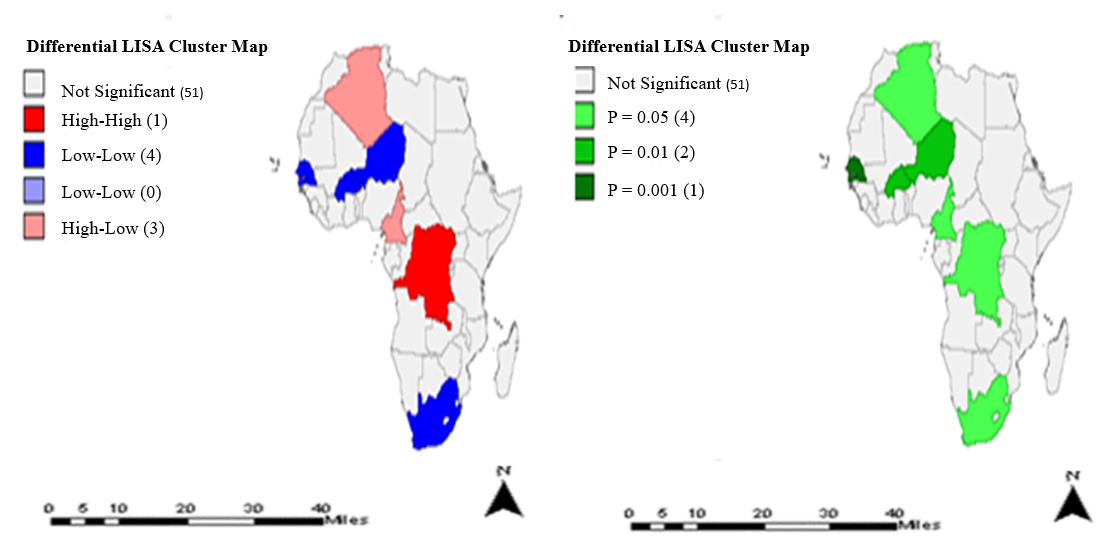 |
